# Structure–Function Relationships in Galactomannan Starch Films for Fast-Dissolving Applications: Effects on Physicochemical and Mechanical Properties

**DOI:** 10.1021/acsomega.6c04905

**Published:** 2026-08-28

**Authors:** Lorena M. F. Sampaio, Matheus X. Oliveira, Venícios G. Sombra, Judith P. A. Feitosa, Nayanne L. dos S. Ferreira, Maria do S. R. Bastos, Sara J. Marciano, Diego Lomonaco, Amélia R. N. Lima, Lara Mota Soares, Crisiana de A. Nobre, Kaliana S. Eça, Thomas Karbowiak, Luciana de S. Oliveira

**Affiliations:** † Departamento de Engenharia de Alimentos, 28121 Universidade Federal do Ceará, Avenida Mister Hull, s/n, Campus Pici, 60455-760 Fortaleza, Ceará, Brasil; ‡ Departamento de Química Orgânica e Inorgânica, Universidade Federal do Ceará, Avenida Mister Hull, s/n, Campus Pici, 60455-760 Fortaleza, Ceará, Brasil; § 196588 Embrapa Agroindústria Tropical, Rua Doutora Sara Mesquita, 2270, Planalto do Pici, 60511-110 Fortaleza, Ceará, Brasil; ∥ Institut Agro, INRAE, UMR PAM (Procédés Alimentaires et Microbiologiques), Bâtiment EPICURE, Université Bourgogne Europe, 1 Esplanade Erasme, 21000 Dijon, France

## Abstract

Orally disintegrating films (ODFs) have attracted increasing attention because of their rapid disintegration and ease of administration. In this study, galactomannan–starch biopolymer films were developed as fast-dissolving oral film matrices using galactomannan extracted from *Caesalpinia pulcherrima* combined with corn and arrowroot (*Maranta arundinacea*) starches at different proportions. The effect of polymer composition on structure–function relationships was systematically investigated, with emphasis on how intermolecular interactions influence the physicochemical and mechanical properties of the films. Starch incorporation significantly affected polymer-network organization, leading to changes in rheological behavior, morphology, and macroscopic performance. Mechanical properties were strongly dependent on starch type: corn starch promoted greater flexibility, whereas arrowroot starch increased rigidity, likely because of differences in amylose content. The films exhibited homogeneous morphology, high transparency, and surface pH values close to neutrality. Water affinity and disintegration behavior were directly influenced by polymer composition, highlighting the role of polymer–polymer interactions in controlling hydration and the structural stability of the developed film matrices. Spectroscopic analysis suggested the presence of intermolecular interactions, particularly hydrogen bonding, which contributed to the modulation of film properties. Among the evaluated formulations, the system containing 75% galactomannan and 25% corn starch exhibited a balanced combination of mechanical strength, flexibility, and rapid disintegration. These findings demonstrate that galactomannan–starch blends are promising materials for ODF applications and provide insights into tailoring structure–function relationships in sustainable biopolymer systems.

## Graphical abstract

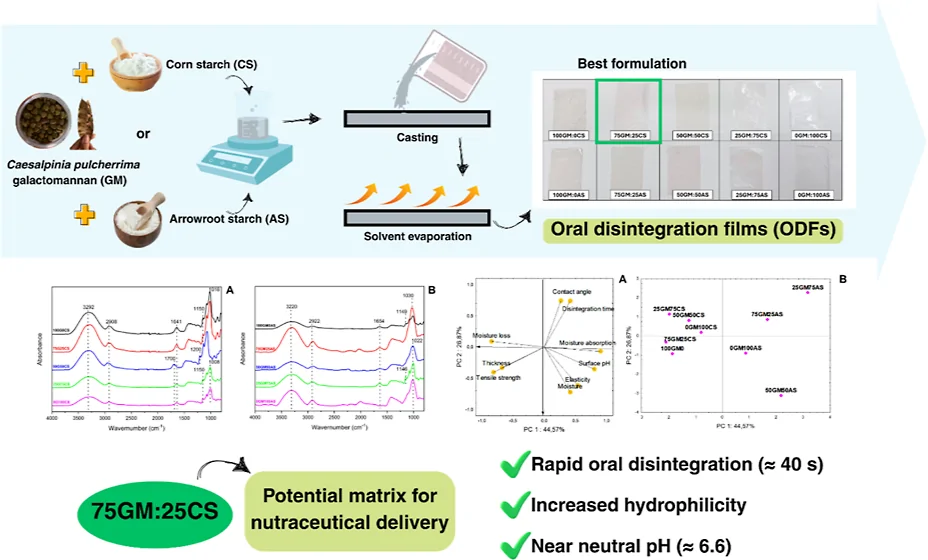

## 1 Introduction

The development of oral delivery systems based on edible films has attracted increasing attention because of their ease of administration, noninvasive nature, and versatility for delivering functional compounds. Fast-dissolving films, commonly referred to as orally disintegrating films (ODFs), have emerged as promising materials for these applications, particularly due to their thin structure, flexibility, and rapid disintegration upon contact with saliva.^1,2^ Typically ranging from 10 to 750 μm in thickness, these films enable the rapid release of incorporated compounds in the oral cavity, and their performance is strongly dependent on the physicochemical properties of the polymeric matrix.^3^

ODFs are generally composed of hydrophilic polymers and plasticizers. Polymers provide the structural framework required for film formation, whereas plasticizers improve flexibility and reduce brittleness during processing and handling.^4^ The selection and combination of polymers are critical factors that influence film properties, including mechanical strength, disintegration behavior, and interaction with water. In this context, polysaccharide-based biopolymers have been widely investigated because of their film-forming ability, biodegradability, and suitability for food-grade applications.^5^

Among polysaccharides, starch is one of the most extensively studied materials for film production because of its availability, low cost, and favorable functional properties. It has been combined with other biopolymers, such as chitosan, gelatin, hydroxypropyl methylcellulose, and carboxymethyl cellulose, to improve film performance.^5–9^ While conventional starch sources, including corn, cassava, wheat, rice, and potato, have been widely investigated, arrowroot starch (*Maranta arundinacea*) remains relatively underexplored. This starch is characterized by a high amylose content and excellent gel-forming ability, which may influence film structure and mechanical behavior.^10^ In addition, arrowroot starch has functional properties that support its application in food and biomaterial systems.^11^

Galactomannans are neutral polysaccharides widely used in food, biomedical, and industrial applications because of their biocompatibility, water solubility, nonionic nature, and ability to form highly viscous dispersions. Structurally, they consist of a β-(1→4)-d-mannan backbone bearing α-(1→6)-linked galactose side chains. Galactomannan extracted from *Caesalpinia pulcherrima*, a species adapted to Brazilian conditions, represents a promising yet underexplored material. Previous studies have reported mannose-to-galactose (M/G) ratios ranging from 2.2 to 3.65, high molar masses, potential for forming structured matrices, and a favorable safety profile.^12–14^

The combination of galactomannans with starches has been investigated as a strategy to tailor the physicochemical and rheological properties of polymeric systems. Interactions between these polysaccharides can influence gelatinization behavior, water affinity, digestibility, and network organization, resulting in materials with distinct functional characteristics.^15,16^ In addition, these interactions may reduce enzymatic degradation and increase resistant starch content, thereby contributing to potential functional benefits. These effects are strongly influenced by structural parameters, such as the mannose-to-galactose ratio of the galactomannan.^17,18^

Despite advances in galactomannan–starch systems, it remains unclear how starch source and proportion affect the structure–function relationships of fast-dissolving films prepared using the same galactomannan matrix. In particular, no systematic comparison has established how the higher amylose content and distinct thermal behavior of arrowroot starch, relative to corn starch, interact with the M/G ratio and high molar mass of *C. pulcherrima* galactomannan to influence solution rheology, polymer-network organization, water interactions, mechanical performance, and disintegration behavior. Therefore, this study systematically compared corn and arrowroot starches at different proportions within a common galactomannan matrix to determine how polymer structure and composition govern processing behavior and the functional performance of the resulting films.

## 2 Materials and Methods

### 2.1 Materials

The following materials were used for film preparation: galactomannan extracted from *C. pulcherrima* seeds collected in Fortaleza, Ceará, Brazil; starch extracted from *M. arundinacea* (arrowroot) tubers, kindly provided by the Centro de Estudos do Trabalho e de Assessoria ao Trabalhador e à Trabalhadora (CETRA; Fortaleza, Ceará, Brazil); and pregelatinized corn starch and sorbitol, both obtained from Adicel Ind. e Com. LTDA (Belo Horizonte, Minas Gerais, Brazil).

### 2.2 Galactomannan Extraction

Galactomannan (GM) was extracted from *C. pulcherrima* seeds according to the method described by Cerqueira et al.,^17^ with minor modifications. The seeds were ground using an industrial blender (LB15, Skymsen), and the endosperm was manually separated from the cotyledons and seed coats. The isolated endosperm was immersed in boiling 50% (v/v) ethanol for 15 min to inactivate endogenous enzymes.

Subsequently, the endosperms were dispersed in distilled water (1:5, w/v) and stored at 8 °C for approximately 12 h. After swelling, they were homogenized with 10 volumes of distilled water using a household blender (LN54, Arno) and filtered through a 150 μm nylon mesh. The resulting viscous solution was precipitated by adding two volumes of absolute ethanol (99.5%, p.a.), followed by immersion of the precipitate in acetone at a ratio of 1:5 (w/v; precipitate/acetone) for 15 min. The precipitate was dried in a forced-air circulation oven at 30 °C for 24 h, ground (MG200, Black + Decker, USA), and sieved through a 0.125 mm mesh. The resulting galactomannan powder was stored in plastic containers in a desiccator containing silica gel at room temperature (25 °C) until further use.

### 2.3 Arrowroot Starch Extraction

Arrowroot starch (AS) was extracted using a wet extraction method as described by Malki et al.^18^ The rhizomes were first cleaned, peeled, and sliced. Mechanical processing consisted of blending the slices with distilled water at a 1:2 (w/v) proportion for 5 min in a stainless steel blender (FAK, LAR-4, Brazil) to produce a uniform paste. The paste was filtered through a 150 μm nylon mesh. This process was repeated three times to maximize yield. After 24 h of sedimentation, the supernatant was manually removed. The starch pellets were dried in an air-circulating oven for 6–8 h at 60 °C, then ground (MG200, Black Decker, USA) and sieved through a 0.125 mm mesh. The resulting starch powder was stored at room temperature (25 °C) until further use.

### 2.4 Characterization of Polysaccharides

#### 2.4.1 Chemical Composition

Moisture content was determined gravimetrically by drying the samples at 105 °C for 3 h and recording the resulting mass loss.^19^ Ash content was determined after complete mineralization of the samples in a muffle furnace at 550 °C for 6 h.^20^ Protein content was determined using the Kjeldahl method,^19^ whereas lipid content was determined by Soxhlet extraction^20^ using analytical-grade *n*-hexane as the solvent. Total carbohydrate content was calculated by difference.

#### 2.4.2 Nuclear Magnetic Resonance (NMR)

Galactomannan (10 mg), arrowroot starch (20 mg), and corn starch (20 mg) were separately dispersed in 700 μL of D_2_O in hermetically sealed containers and stirred at 70 °C for 24 h. The sealed containers were used to minimize solvent evaporation during heating. After this period, the samples were cooled to room temperature, and the volume was adjusted to the initial value of 700 μL with D_2_O before transfer to 5 mm NMR tubes. Proton nuclear magnetic resonance (^1^H NMR) spectra were acquired using a Bruker Avance DRX-500 spectrometer operating at 500 MHz and equipped with a 5 mm dual probe. Measurements were performed at 70 °C using DSS (2,2-dimethyl-2-silapentane-5-sulfonic acid) as the internal standard.

For GM, the anomeric proton signals between 4.6 and 5.2 ppm were integrated, and the mannose-to-galactose (M/G) ratio was calculated from their relative areas. The spectra of CS and AS were used only for structural confirmation (Figure S1), with signals observed between 1.5 and 6.0 ppm.

#### 2.4.3 Amylose Content

The amylose content of the starches was determined according to the method described by Martinez and Cuevas,^21^ with modifications proposed by Nogueira et al.^22^ AS and CS samples (10 mg each) were mixed with 0.1 mL of 95% ethanol and 0.9 mL of 1 mol L^–1^ NaOH and then heated in a boiling-water bath at 100 °C for 5 min. After cooling, the solutions were diluted to a final volume of 10 mL with distilled water. A 0.5 mL aliquot was mixed with 0.2 mL of 0.2% iodine solution and 0.1 mL of 1 mol L^–1^ acetic acid. The mixture was then diluted to a final volume of 10 mL, incubated in the dark for 20 min, and its absorbance was measured at 625 nm using a UV–Vis spectrophotometer (model 1800, Shimadzu, Japan).

Amylose content was quantified using the corresponding calibration curve over the range of 0.12–1.00 mg mL^–1^, according to the equation *y* = 1.209*x* + 0.023*y* = 1.209*x* + 0.023*y* = 1.209*x* + 0.023, where *y* represents absorbance and *x* represents amylose concentration in mg mL^–1^.

#### 2.4.4 Solubility

Polysaccharide solubility was determined according to the method described by Malki et al.,^18^ with modifications. GM (0.1 g) was dispersed in 20 mL of distilled water, whereas AS and CS samples (0.25 g each) were separately dispersed in 10 mL of distilled water. After magnetic stirring at 78 °C for 30 min, the dispersions were centrifuged at 3000 rpm for 15 min. The supernatants were transferred to preweighed glass Petri dishes and dried in an oven at 130 °C for 2 h. After cooling, the dishes were weighed again. Solubility was calculated according to eq 1, where *W*_sp_ is the mass of soluble polysaccharide and *W*_s_ is the initial sample mass.

1 $$solubility\;\left(\%\right)=\left(W_{sp}/W_{s}\right)\times 100$$

#### 2.4.5 Gel Permeation Chromatography (GPC)

The peak molar mass *M*_pk_ of GM, CS, and AS was determined by gel permeation chromatography (GPC) using a Shimadzu LC-20CE system equipped with an RID-10A refractive index detector and a linear PolySep column (7.8 × 300 mm). The mobile phase consisted of 0.1 mol L^–1^ NaNO_3_, delivered at a flow rate of 1 mL min^–1^, and the injection volume was 50 μL. Calibration was performed using pullulan standards (Shodex Denko) covering a molar mass range of 10^3^ to 10^6^ g mol^–1^.

For CS and AS analysis, the pH of the mobile phase was adjusted to 11.5 using 1 mol L^–1^ NaOH. Before injection, the samples were dissolved in deionized water at a concentration of 1 mg mL^–1^, and the pH of the CS and AS solutions was also adjusted to 11.5 with 1 mol L^–1^ NaOH. All polysaccharide solutions were filtered through a 0.20 μm membrane before chromatographic analysis.

#### 2.4.6 Differential Scanning Calorimetry (DSC)

DSC thermograms of the polysaccharides were obtained using a differential scanning calorimeter (DSC823e, Mettler-Toledo, Switzerland). Approximately 5 mg of each sample was sealed in an aluminum crucible with a perforated lid and analyzed under a nitrogen atmosphere at a flow rate of 50 mL min^–1^. The samples were heated from 25 to 100 °C at a heating rate of 5 °C min^–1^.

#### 2.4.7 Thermogravimetric Analysis (TGA)

Thermogravimetric analysis (TGA) of the isolated polysaccharides (GM, CS, and AS) was performed using a TA Instruments Q50 thermogravimetric analyzer under a nitrogen atmosphere at a flow rate of 60 mL min^–1^. The samples were heated from 25 to 800 °C at a heating rate of 10 °C min^–1^.

### 2.5 Preparation of Oral Disintegrating Films

The films were prepared using two formulation sets based on different GM/starch ratios. In the first set, the proportions of GM and CS were varied, whereas in the second set, the proportions of GM and AS were varied. This resulted in nine formulations: three control films composed exclusively of GM, CS, or AS; three GM/CS formulations at ratios of 75:25, 50:50, and 25:75; and three GM/AS formulations at the same ratios (Table 1).

**Table 1:** Formulations of Galactomannan (GM) and Corn Starch (CS) or Arrowroot Starch (AS) Used for Film Preparation

formulations	GM/CS		formulations	GM/AS	
	GM (%)	CS (%)		GM (%)	AS (%)
100GM:0	100	0	100GM:0	100	0
75GM:25CS	75	25	75GM:25AS	75	25
50GM:50CS	50	50	50GM:50AS	50	50
25GM:75CS	25	75	25GM:75AS	25	75
0GM:100CS	0	100	0GM:100AS	0	100

To prepare the films, a total polysaccharide concentration of 1% (w/v), corresponding to 1 g of polysaccharides per 100 mL of distilled water, was used according to the proportions listed in Table 1. GM was gradually added to distilled water under magnetic stirring at room temperature (25 °C) to prevent lump formation. After complete dissolution, the starch was added, and the mixture was stirred at 25 °C for 1 h. The temperature was then increased to 90 °C and maintained for 30 min. After cooling the dispersion to approximately 30 °C, sorbitol was added at 0.4 g per gram of polysaccharide.^23,24^ The film-forming solutions were allowed to rest at 25 °C for 24 h to facilitate the removal of residual air bubbles.

A 100 mL aliquot of each film-forming solution was cast onto acrylic plates (20 × 20 cm) and dried in a forced-air oven (MA 035, Marconi, Brazil) at 40 °C for 20–24 h. After drying, the films were removed from the plates and conditioned in a desiccator containing a saturated sodium bromide solution at 58% relative humidity and 25 °C for 48 h before testing.

### 2.6 Rheological Characterization of the Film-Forming Solutions

The rheological behavior of the film-forming solutions containing different GM/CS and GM/AS ratios was evaluated using a rotational rheometer equipped with concentric cylinders and a DG-DIN sensor (R/S Plus SST 2000, Searle, Brookfield, Canada). The shear rate was increased from 0 to 200 s^–1^ and subsequently decreased from 200 to 0 s^–1^ to obtain the ascending and descending flow curves, respectively. Each curve was recorded over 1 min using 25 data points at 25 °C. Shear stress and shear rate data were acquired using the RHEO V 2.8 software supplied with the instrument.

### 2.7 Characterization of the Films

#### 2.7.1 Film Thickness and Moisture Content

Film thickness was measured using a digital micrometer (QuantuMike, Mitutoyo, USA). Measurements were taken at five different locations on each film, and the results were expressed in micrometers (μm). Moisture content was determined from the mass difference before and after drying at 105 °C for 3 h, according to AOAC.^19^

#### 2.7.2 Oral Disintegration Time

The in vitro disintegration time of the films was determined according to the method described by Garsuch and Breitkreutz.^25^ Film specimens (3.0 × 2.0 cm) were placed in Petri dishes, and 200 μL of distilled water was deposited onto the surface of each film. The in vitro disintegration time was defined as the time elapsed until water penetration caused film perforation. This method was used as a comparative screening test among the formulations and was not intended to reproduce the full physiological conditions of the oral cavity.

#### 2.7.3 Surface pH

The surface pH of the films was determined according to the procedure described by Thombre and Gide.^26^ A phosphate buffer solution at pH 6.8, used to simulate human saliva, was prepared according to Föger et al.^27^ using 2.38 g of Na_2_HPO_4_, 0.19 g of KH_2_PO_4_, and 8.00 g of NaCl per liter of distilled water. The final pH was adjusted with phosphoric acid.

Film specimens (3.0 × 2.0 cm) were immersed in 1000 μL of buffer and left undisturbed for 1 min. The pH electrode was then placed in contact with the film surface for 1 min, and the resulting values were recorded.

#### 2.7.4 Colorimetry and Transparency

The color parameters of the film specimens were measured using a ColorQuest XE colorimeter (HunterLab, USA) equipped with a reflectance sensor. Each specimen was cut to fit a 1 cm-thick cuvette with a measurement area of 5.31 cm^2^. Color was expressed using the following parameters: L*, corresponding to lightness from 0 (black) to 100 (white); a*, ranging from green (−) to red (+); b*, ranging from blue (−) to yellow (+); and C*, corresponding to chroma, with higher values indicating greater color saturation.

Transparency was evaluated using a UV–Vis spectrophotometer (model 1800, Shimadzu, Japan) at 600 nm. The transparency index normalized by film thickness was calculated according to eq 2

2 $$transparency=\left(Abs_{600nm}/y\right)\times 100$$

where Abs_600_ is the absorbance at 600 nm and *y* is the film thickness (μm). According to this equation, higher transparency index values correspond to lower film transparency.

#### 2.7.5 Mechanical Properties of the Films

Tensile strength and elongation at break were determined using a TA.XTplus texture analyzer (Stable Micro Systems, Godalming, Surrey, UK) according to ASTM D882-10 (2010). Ten film specimens (5.0 × 2.5 cm) were individually mounted in A/TG tensile grips. Tensile stress and elongation at break were obtained from the stress–strain curves. Tensile strength was calculated using the measured thickness of each individual specimen.

#### 2.7.6 Water Contact Angle

Water contact angle measurements were performed to assess film surface wettability. Film specimens (5.0 × 2.5 cm) were fixed onto glass slides using double-sided tape. A distilled water droplet (∼7 μL) was deposited onto the film surface using an automatic pipet, and droplet images were captured using a Desidrop camera (DGD-MCAT, GBX, Dublin, Ireland). Five measurements were performed for each formulation. Contact angle values were determined from images acquired immediately after droplet deposition.

#### 2.7.7 Moisture Loss and Moisture Uptake

Film specimens (2.0 × 3.0 cm) were accurately weighed and placed in desiccators containing supersaturated calcium chloride solution (RH ≈ 30%) at room temperature for 72 h. The specimens were then reweighed, and moisture loss was calculated according to eq 3

3 lossmoisture(%)=[(initialmass−finalmass)/(initialmass)]×100

For moisture uptake determination, film specimens (2.0 × 3.0 cm) were placed in desiccators containing saturated potassium sulfate solution (RH ≈ 75%) at room temperature for 24 h. Moisture uptake was calculated according to eq 4

4 moistureabsorption(%)=[(finalmass−initialmass)/(initialmass)]×100

#### 2.7.8 Fourier Transform Infrared Spectroscopy (FTIR)

FTIR spectra were recorded using a PerkinElmer Spectrum Two FTIR spectrometer (PerkinElmer, USA) equipped with an attenuated total reflectance (ATR) accessory. The spectra were collected over the wavenumber range of 4000–400 cm^–1^.

#### 2.7.9 Scanning Electron Microscopy (SEM)

The surface morphology of the films was examined by scanning electron microscopy (SEM) using an Inspect S50-FEI microscope (FEI, Oregon, USA). The specimens were sputter-coated with a 35 nm gold–palladium (Au–Pd) layer and analyzed at an accelerating voltage of 10 kV.

### 2.8 Statistical Analysis

All analyses were performed in triplicate. Differences among mean values were assessed using Tukey’s test at a significance level of 5% (*p* < 0.05). Principal component analysis (PCA) was also performed to explore relationships among the physicochemical and mechanical properties of the films. The analysis was conducted using a standardized data matrix, and principal components were retained based on eigenvalues greater than 1 and cumulative explained variance. This multivariate approach enabled the identification of the variables that contributed most to differences among the formulations. Statistical analyses were performed using Statistica 10.0 (Dell, Texas, USA) and OriginPro 8.5 (OriginLab Corporation, Massachusetts, USA).

## 3 Results and Discussion

### 3.1 Chemical Characteristics of Extracted Polysaccharides

The extraction yield of arrowroot starch (AS) averaged 16%, which was higher than the 12% reported by Malki et al.^18^ Although the extraction procedure was similar to that described by these authors, factors such as climatic conditions and rhizome maturity at harvest may influence extraction yield. More mature rhizomes generally contain higher starch contents.^10,18^ In contrast, the extraction yield of GM from *C. pulcherrima* seeds was slightly lower than values reported in the literature. The present study obtained a yield of 17%, whereas Cerqueira et al.^17^ reported an average yield of 25%.

Knowledge of the chemical composition and purity of the materials is essential for their application in film development. Table 2 presents the chemical composition of the extracted polysaccharides, including moisture, ash, lipid, protein, and carbohydrate contents, which were consistent with values reported in the literature.^18,22,26^

**Table 2:** Chemical Composition of the Extracted Corn Starch (CS), Arrowroot Starch (AS), and Galactomannan (GM)^a^

chemical composition	samples		
	CS	AS	GM
moisture (%)	10.82 ± 0.15^b^	9.04 ± 0.02^c^	11.77 ± 0.15^a^
ash (%)	0.99 ± 0.00^b^	0.99 ± 0.00^c^	1.00 ± 0.00^a^
lipids (%)	1.82 ± 0.01^b^	1.31 ± 0.01^c^	3.66 ± 0.51^a^
proteins (%)	0.03 ± 0.01^a^	0.02 ± 0.00^b^	0.03 ± 0.00^a^
carbohydrates (%)	86.34 ± 0.04^b^	90.87 ± 0.03^a^	83.54 ± 0.17^c^
amylose (%)	28.90 ± 0.90^b^	35.24 ± 1.63^a^	-
M/G	-	-	2.53:1

a Different letters within the same row indicate significant differences according to Tukey’s test (*p* < 0.05).

The amylose content of AS (35.24 ± 1.63%) was higher than the value of 24.95 ± 1.49% reported by Malki et al.,^18^ likely because of differences in plant maturity. Tarique et al.^10^ reported that the amylose content of AS may range from 40 to 45%, whereas that of CS typically ranges from 25 to 30%. The mechanical behavior of the films is strongly influenced by amylose content because the linear structure of amylose favors tighter molecular packing than branched amylopectin, thereby contributing to improved tensile and barrier properties.^28^

For GM, one of the key structural parameters relevant to film formulation is the mannose-to-galactose (M/G) ratio, which was determined by ^1H NMR analysis (Figure 1). In the spectrum, the signals at δ 4.74 and 5.02 ppm were assigned to the anomeric protons of β-d-mannopyranose and α-d-galactopyranose residues, respectively.^29^

**Figure 1:**
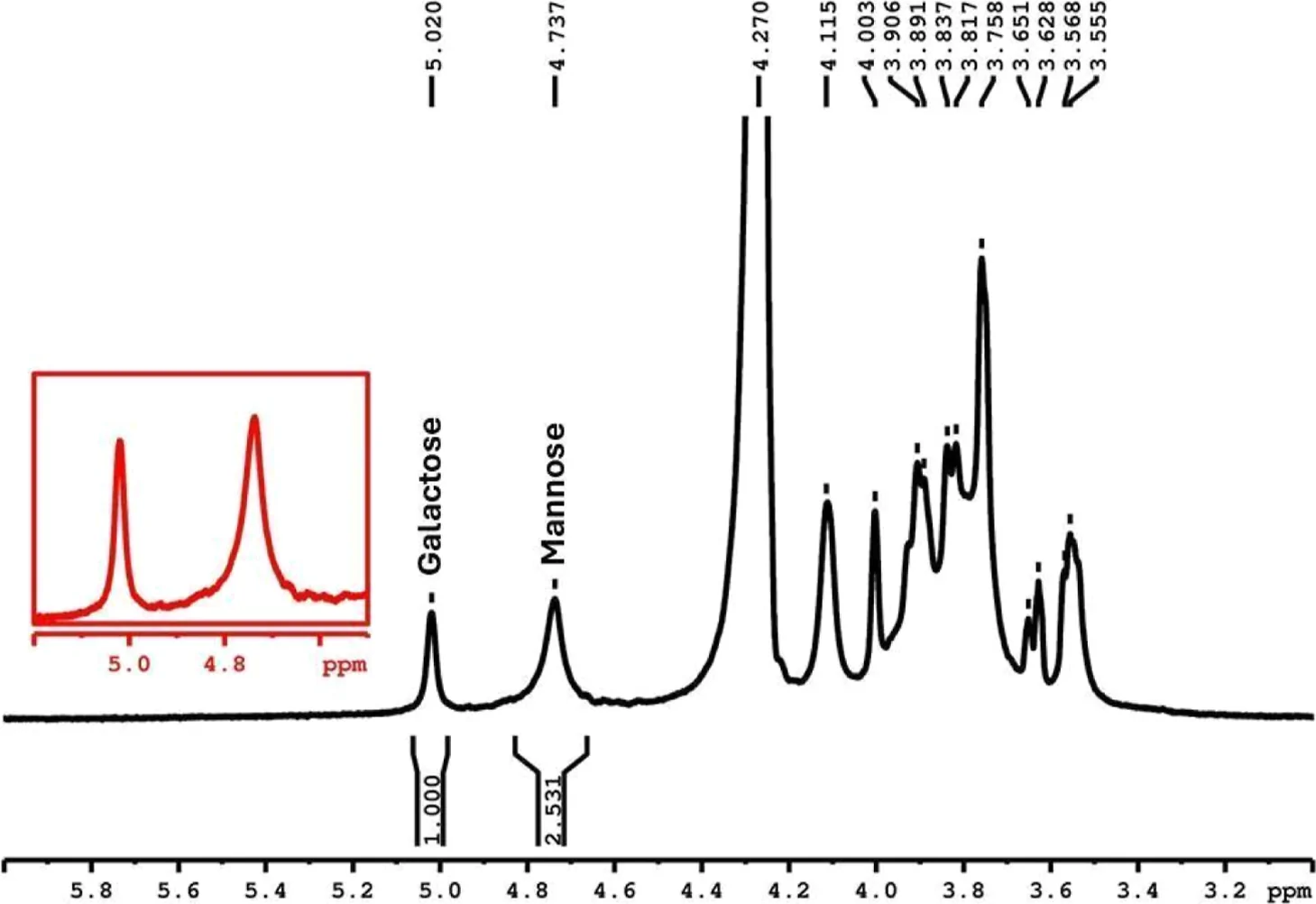
^1^H NMR spectrum (D_2_O, 70 °C, 500 MHz) of galactomannan isolated from *C. pulcherrima*. The anomeric signals at δ 5.02 and 4.74 ppm correspond to α-d-galactopyranose and β-d-mannopyranose, respectively. The relative integration values (1.000 and 2.531) were used to calculate the M/G ratio of 2.53:1.

The M/G ratio was calculated from the relative integrated areas of the signals assigned to the identified monosaccharide residues and was found to be 2.53:1. This value was intermediate between those reported by Thombre and Gide^26^ (2.80:1) and da Da Cunha Jácome Marques et al.^12^ (2.18:1) for galactomannans extracted from *C. pulcherrima*. According to Buriti et al.,^30,31^ the M/G ratio may range from 1.1 to 5.0 depending on factors such as plant species, pod maturity, and extraction method.

Prajapati et al.^32^ reported that galactomannans with a higher galactose content are more water-soluble and less prone to gel formation because the greater number of galactose side chains keeps mannose residues sufficiently separated, thereby limiting strong intermolecular interactions. The galactomannan extracted from *C. pulcherrima* seeds exhibited a relatively lower galactose content and higher mannose content, suggesting that its less-substituted mannan backbone may favor interactions with other polysaccharides.

The peak molar mass (*M*_pk_) and solubility values of CS, AS, and GM are summarized in Table 3. The *M*_pk_ values obtained for CS and AS likely correspond predominantly to their soluble amylose fractions, consistent with the methodological considerations described by Chen et al.^33^

**Table 3:** Peak Molar Mass (*M*_pk_) and Solubility of CS, AS and GM^a^

samples	*M*_pk_ (g mol^–1^)	solubility (%)
CS	1.42 × 10^3^*	15.66 ± 0.47^c^
AS	1.74 × 10^3^*	48.98 ± 0.62^a^
GM	5.23 × 10^6^	24.48 ± 1.51^b^

a Different letters in the column referring to solubility analysis show significant difference (*p* < 0.05) by Tukey’s test. * not applicable. * The *M*_pk_ values comprise only the amylose fractions of the polymer.

The M_pk_ value determined for GM was close to that reported by da Da Cunha Jácome Marques et al.^12^ for a sample previously isolated in Ceará, Brazil (2.82 × 10^6^ g mol^–1^). In contrast, GM isolated in São Paulo, Brazil, exhibited a lower molar mass of 1.01 × 10^6^ g mol^–1^.^34^ These differences indicate that the molar mass of plant-derived polysaccharides may vary according to factors associated with geographical origin and raw-material characteristics.

Because of its high *M*_pk_, GM is expected to form highly viscous hydrocolloidal solutions. Similarly, Oliveira et al.^29^ reported high viscosity for commercial galactomannan (guar gum) with a molar mass of 5.3 × 10^6^ g mol^–1^, which is comparable to that estimated for GM in the present study.

Solubility is associated with starch composition, particularly amylose content. As shown in Table 3, AS exhibited a significantly higher solubility (48.98 ± 0.62%) than CS (15.66 ± 0.47%). This greater solubility may be related to the higher amylose content of AS, since the leaching and mobilization of low-molar-mass amylose from amorphous regions during heating can promote starch solubilization.^18,35^

### 3.2 Thermal Properties of Extracted Polysaccharides

Figure 2 presents the thermogravimetric (TGA), derivative thermogravimetric (DTG), and differential scanning calorimetry (DSC) curves of CS, AS, and GM.

**Figure 2:**
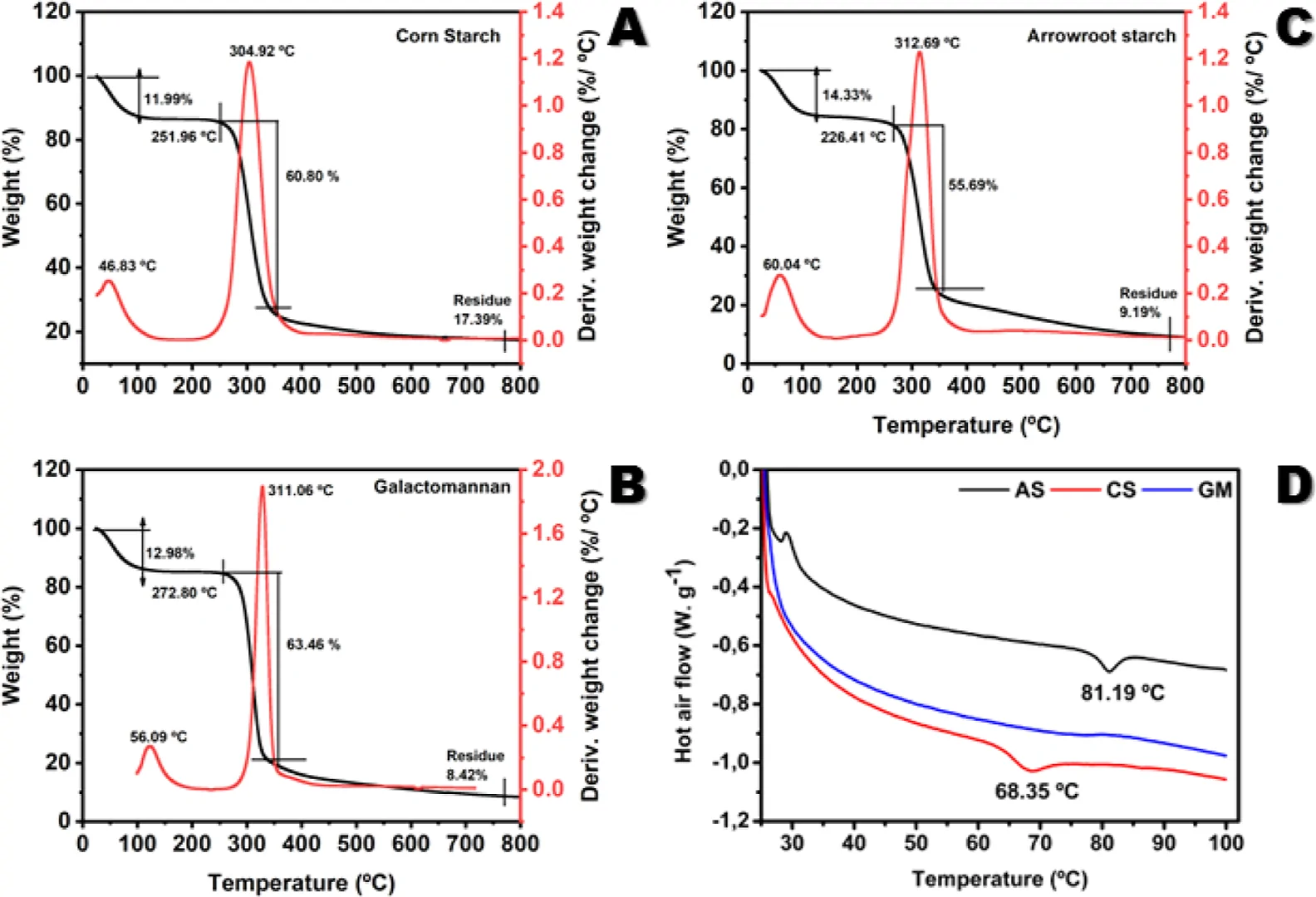
Thermogravimetric (TGA/DTG) curves of corn starch (A), arrowroot starch (B), and galactomannan (C), and differential scanning calorimetry (DSC) thermograms of these polysaccharides (D).

The TGA profiles revealed two distinct mass-loss stages for all three polysaccharides. The first stage, with a maximum mass-loss rate temperature (*T*_max_) of approximately 46–60 °C, was attributed to moisture removal and accounted for mass losses of 12.0%, 13.0%, and 14.3% for CS, GM, and AS, respectively. The second stage occurred between 250 and 350 °C and was associated with depolymerization and thermal degradation of the polysaccharide chains, including amylose and amylopectin in the starch samples. This event exhibited *T*_max_ values of 304.9, 311.1, and 312.7 °C, with corresponding mass losses of 60.8%, 55.7%, and 63.5% for CS, GM, and AS, respectively. Differences in degradation temperature among starches from different botanical sources may be related to granule size, microstructure, crystalline organization, and the amylose-to-amylopectin ratio.^36^ Because these degradation temperatures were substantially higher than the 90 °C used to prepare the film-forming solutions, thermal degradation of GM, CS, and AS was not expected during processing.

The DSC curves (Figure 2D) were used to evaluate the thermal behavior of the polysaccharides under conditions relevant to film formation. The analysis was conducted over the temperature range of 0–100 °C. Only the starches exhibited endothermic transitions. CS showed an onset temperature of 63.6 °C and a peak temperature of 68.4 °C, whereas AS exhibited higher onset and peak temperatures of 77.7 and 81.1 °C, respectively (Table S1), indicating greater resistance to disruption of its ordered structure.

The higher transition temperature observed for AS suggests greater resistance to disruption of its ordered structure during heating, which may be associated with its higher amylose content and distinct molecular organization. This difference is relevant to film processing because the film-forming dispersions were heated at 90 °C for 30 min. Because this processing temperature exceeded the thermal transitions of both starches, it was expected to promote extensive gelatinization and molecular dispersion. However, GM may affect starch swelling and gelatinization by competing for available water, increasing the viscosity of the continuous phase, and forming intermolecular interactions with starch chains. These effects may contribute to the distinct rheological behavior and structural organization observed in the GM/CS and GM/AS systems.

No endothermic event was observed for GM within the evaluated temperature range, as its melting temperature has been reported to lie between 230 and 300 °C. Unlike starch, GM does not undergo gelatinization but instead hydrates and dissolves in aqueous media. Because the DSC analysis was intended to assess thermal events relevant to the processing conditions used to prepare the film-forming solutions, higher temperature ranges were not investigated.

### 3.3 Rheology of the Film-Forming Solutions

Determining the rheological behavior of film-forming solutions is important because viscosity and flow characteristics can influence spreading, leveling, bubble removal, and film uniformity during casting.^37,38^ The experimental data were fitted to the Ostwald–de Waele (ODW) and Herschel–Bulkley (HB) models, both of which yielded *R* ^2^ values above 0.95. The ODW model was applied to formulations without measurable yield stress, whereas the HB model was used for 50GM:50CS and 25GM:75AS, which exhibited nonzero yield stress. The model assigned to each formulation is indicated in Table 4.

**Table 4:** Initial Yield Stress (τ_0_), Consistency Index (*K*), Flow Behavior Index (*n*), and Coefficient of Determination (*R* ^2^) Obtained by Fitting the Ostwald–de Waele and Herschel–Bulkley Models to Film-Forming Solutions Containing Different GM/CS and GM/AS Proportions^a^

samples	model	τ_0_ (Pa)	*K* (Pa × s^*n*^)	*n*	*R* ^2^
100GM:0	ODW	NS	9.727 ± 0.159^a^	0.433 ± 0.006^d^	0.999
75GM:25CS	ODW	NS	3.300 ± 0.061^c^	0.563 ± 0.006^d^	0.999
75GM:25AS	ODW	NS	5.331 ± 0.039^b^	0.493 ± 0.001^d^	0.994
50GM:50CS	HB	3.612 ± 0.139^a^	0.069 ± 0.004^e^	1.216 ± 0.003^c^	0.991
50GM:50AS	ODW	NS	1.000 ± 0.002^d^	0.712 ± 0.004^d^	0.998
25GM:75CS	ODW	NS	0.007 ± 0.003^e^	1.513 ± 0.030^c^	0.997
25GM:75AS	HB	0.807 ± 0.036^b^	0.008 ± 0.004^e^	1.526 ± 0.060^c^	0.996
0GM:100CS	ODW	NS	3.910 × 10^–4^ ± 2.410 × 10^–4e^	2.347 ± 0.332^a^	0.964
0GM:100AS	ODW	NS	3.393 × 10^–4^ ± 4.19 × 10^–4e^	1.913 ± 0.193^b^	0.994

a Different letters within the same column indicate significant differences among the film-forming solutions according to Tukey’s test (*p* < 0.05). NS: no measurable yield stress; ODW: Ostwald–de Waele model; HB: Herschel–Bulkley model.

The formulations with higher GM concentrations exhibited flow behavior index values (*n*) below 1, confirming pseudoplastic behavior, whereas lower GM concentrations, corresponding to higher starch contents, resulted in *n* values above 1, indicating dilatant behavior. Decreasing the GM concentration also reduced the consistency index (*K*), suggesting a less structured polymeric system.^23^ However, amylose content may not be the only factor involved. The higher thermal transition temperature of AS, its stronger water-binding capacity, and the possible persistence of swollen starch structures may also contribute to the greater consistency of the GM/AS systems.

As shown in Table 3, GM exhibited the highest *M*_pk_, 5.23 × 10^6^ g mol^–1^, compared with 1.42 × 10^3^ and 1.74 × 10^3^ g mol^–1^ for CS and AS, respectively, which is consistent with its higher *K* value. Although the starches exhibited similar *M*_pk_, AS had a higher amylose content (Table 2), which may have contributed to the greater consistency observed during preparation of the film-forming solutions.^18,35^ This higher consistency may improve film stability after casting, although excessively viscous solutions can hinder leveling and bubble removal.

Buriti, dos Santos et al.^30^ and Buriti, Freitas et al.^31^ reported pseudoplastic behavior in 1% GM solutions, which is the same polysaccharide concentration used in the 100GM:0 formulation. In contrast, Tedesco et al.^23^ study evaluating film-forming solutions containing 5% starch also described dilatant behavior, consistent with the results of the present work.

From a processing perspective, pseudoplastic behavior may facilitate spreading because apparent viscosity decreases under shear. After shear removal, viscosity recovery may help limit excessive flow and promote thickness uniformity. Conversely, excessively low viscosity may lead to uneven spreading, whereas excessively high viscosity may hinder bubble migration and surface leveling.^39^

Figure 3A,B show that increasing the CS or AS concentration while decreasing the GM concentration resulted in more linear flow curves, indicating behavior closer to that of a Newtonian fluid. Consistent with this observation, Figure 3C,D show that the 50GM:50CS, 50GM:50AS, 25GM:75CS, 25GM:75AS, 0GM:100CS, and 0GM:100AS formulations exhibited smaller changes in viscosity with increasing shear rate. In contrast, formulations with higher GM contents showed a marked decrease in viscosity as the shear rate increased, which is characteristic of pseudoplastic behavior.

**Figure 3:**
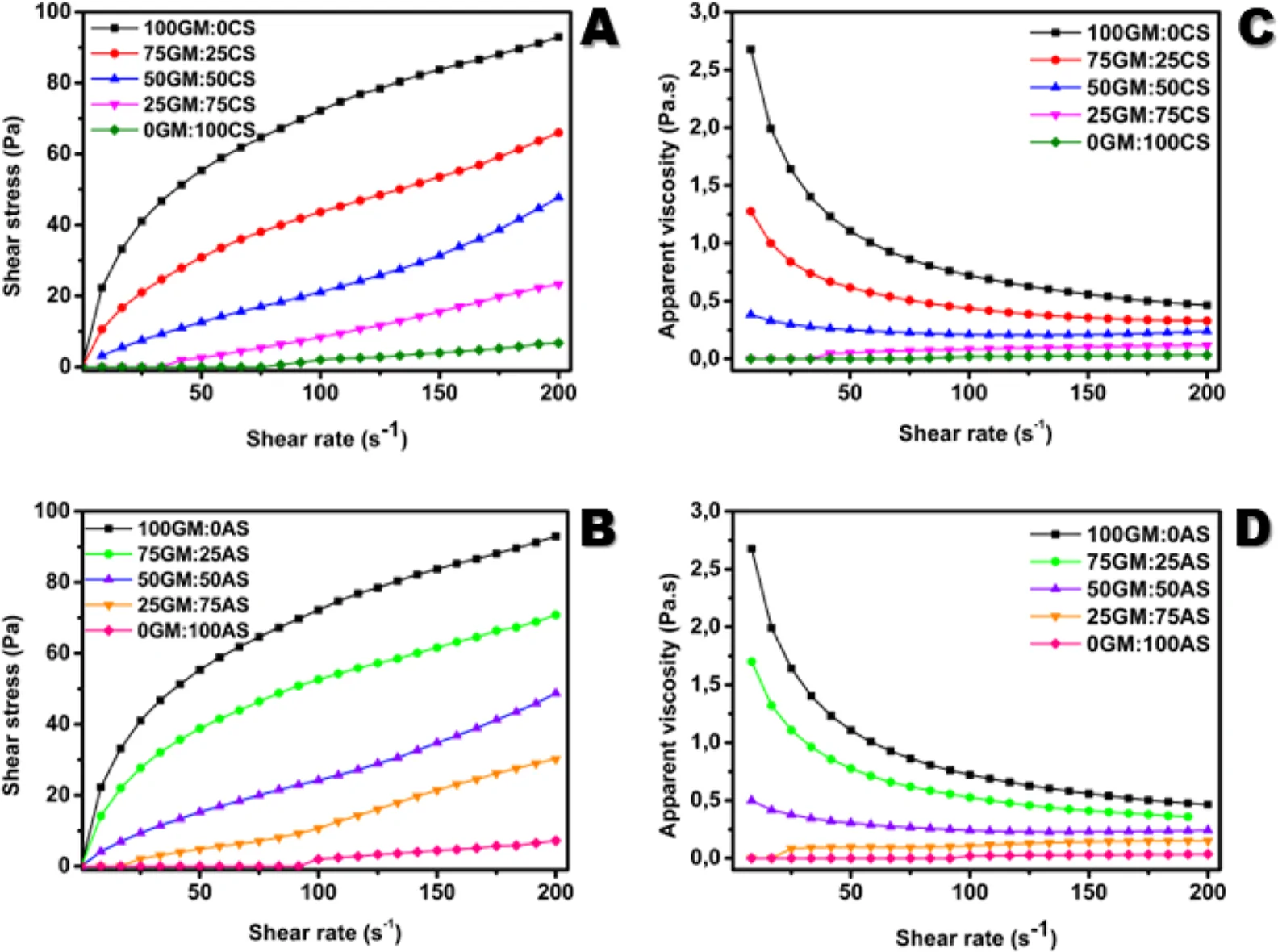
Flow curves (A,B) and apparent viscosity curves (C,D) of film-forming solutions prepared with different GM/CS and GM/AS ratios.

### 3.4 Visual Aspect of Film and Colorimetric Analysis

Figure 4 shows the films prepared with different proportions of GM, CS, and AS. All formulations produced transparent and homogeneous films, with no visible air bubbles and a uniform appearance throughout the specimens. These characteristics are commonly reported for films based on CS, AS, and GM.^10,23,24,40,41^ From the perspective of oral-film applications, the absence of visible defects and air bubbles is relevant because such imperfections may compromise dose uniformity and visual acceptability. Although high transparency is not an essential requirement for ODFs, a clearer appearance facilitates the visual assessment of film homogeneity and surface defects.^42,43^

**Figure 4:**
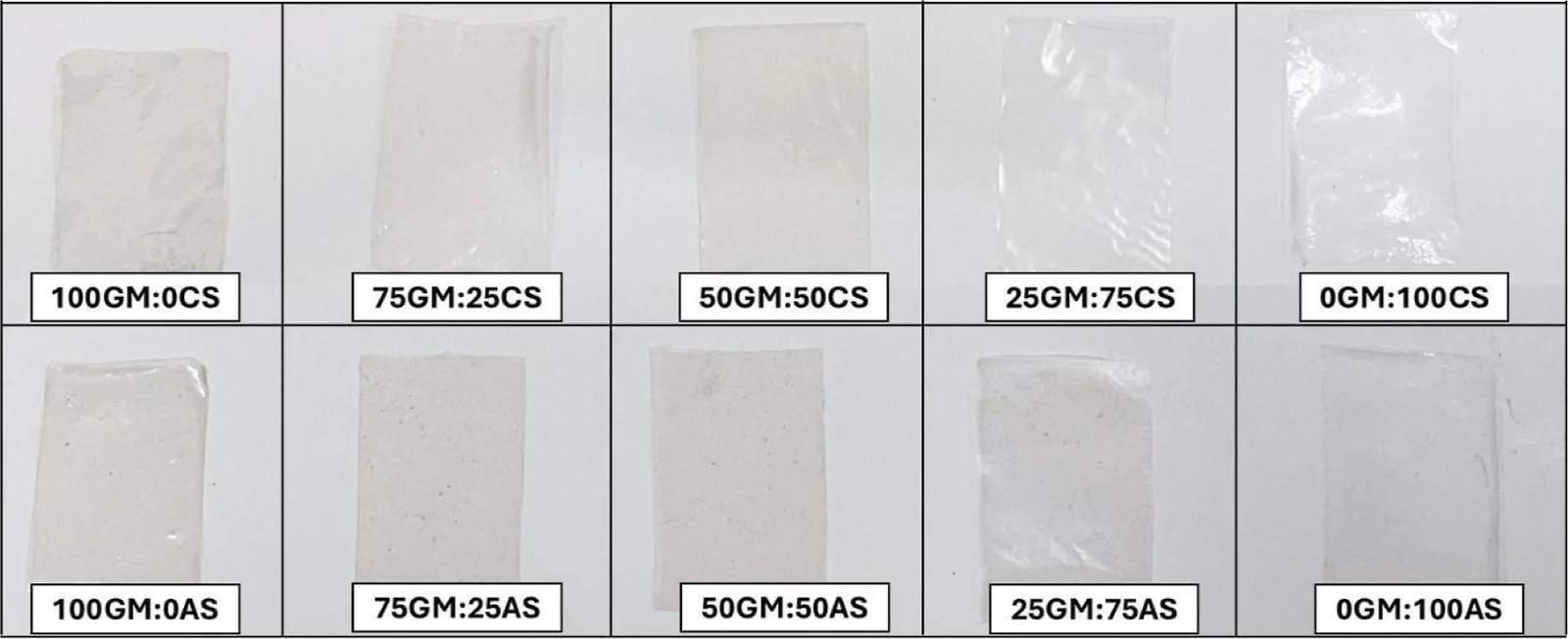
Films prepared with different GM/CS and GM/AS ratios. The films were cut into rectangular specimens measuring 3 × 2 cm.

However, films prepared with GM and CS were easily removed from the acrylic plates, whereas those containing GM and AS were more difficult to remove and exhibited a slightly sticky surface, except for the formulation composed solely of AS. This behavior may be related to differences in polymer wettability, residual moisture retention, and network consolidation during drying. The stickiness observed in some formulations may also result from interactions among AS, GM, water, and sorbitol, although this property was not quantitatively evaluated in the present study. From a technological perspective, difficult removal from the casting plate may impair demolding, cutting, handling, packaging, and process scale-up, thereby limiting the suitability of these formulations as ODF matrices.

The visual appearance and colorimetric data presented in Figure 4 and Table 5, respectively, showed high lightness (L*) and low chroma (C*) values for the films, consistent with their clear appearance. Color and transparency may contribute to consumer acceptability and overall product appearance, although transparency is not a functional requirement for all ODF applications.^2,44^

**Table 5:** Colorimetric Parameters of Films Prepared With Different GM/CS and GM/AS Ratios^a^

samples	a*	b*	L*	C*	transparency index (*A*_600_ × 100 μm^–1^)
100GM:0	1.37 ± 0.08^ab^	–2.51 ± 0.06^a^	63.71 ± 0.36^ab^	2.86 ± 0.07^a^	2.03 ± 0.20^b^
75GM:25CS	1.55 ± 0.03^a^	–2.72 ± 0.01^a^	61.00 ± 0.06^b^	3.14 ± 0.02^a^	1.96 ± 0.07^b^
75GM:25AS	1.41 ± 0.01^ab^	–2.74 ± 0.04^a^	61.38 ± 0.32^b^	3.08 ± 0.04^a^	1.76 ± 0.09^b^
50GM:50CS	1.39 ± 0.05^ab^	–2.52 ± 0.01^a^	62.09 ± 0.09^ab^	2.88 ± 0.04^a^	1.17 ± 0.19^c^
50GM:50AS	1.38 ± 0.16^ab^	–2.75 ± 0.18^a^	62.50 ± 0.12^ab^	3.08 ± 0.23^a^	0.36 ± 0.06^d^
25GM:75CS	1.42 ± 0.02^ab^	–2.77 ± 0.01^a^	61.61 ± 0.09^ab^	3.11 ± 0.01^a^	1.97 ± 0.05^b^
25GM:75AS	1.53 ± 0.01^a^	–2.73 ± 0.43^a^	63.63 ± 2.75^ab^	3.05 ± 0.50^a^	0.18 ± 0.03^d^
0GM:100CS	1.11 ± 0.01^c^	–2.37 ± 0.06^a^	64.35 ± 0.16^a^	2.61 ± 0.06^a^	2.65 ± 0.26^a^
0GM:100AS	1.27 ± 0.10^bc^	–2.65 ± 0.11^a^	62.60 ± 0.81^ab^	2.94 ± 0.14^a^	1.11 ± 0.17^c^

a Different letters within the same column indicate significant differences among the films according to Tukey’s test (*p* < 0.05).

The transparency index of the films (Table 5) ranged from 0.18 to 2.65 A_600_ μm^–1^. The film composed solely of CS exhibited the highest transparency index (2.65 A_600_ μm^–1^) and was therefore considered the least transparent. In contrast, films containing AS generally showed lower transparency index values, particularly the 50GM:50AS (0.36 A_600_ μm^–1^) and 25GM:75AS (0.18 A_600_ μm^–1^) formulations, indicating greater optical transparency.

### 3.5 Surface pH, Mechanical Properties, Oral Disintegration Time, and Water Contact Angle

Table 6 summarizes the surface pH, tensile strength, elongation at break, disintegration time, and water contact angle of the films.

**Table 6:** Surface pH, Mechanical Properties, Disintegration Time, and Water Contact Angle of Films Prepared with Different GM/CS and GM/AS Ratios^a^

samples	surface pH	Tensile strength (MPa)	elasticity (%)	disintegration time (s)	contact angle (°)
100GM:0	6.68 ± 0.07^cd^	16.19 ± 0.71^a^	18.58 ± 4.69^b^	22.00 ± 2.65^f^	49.65 ± 4.62^b^
75GM:25CS	6.57 ± 0.11^d^	12.69 ± 2.08^ab^	35.69 ± 9.84^a^	41.00 ± 4.36^e^	49.61 ± 5.47^b^
75GM:25AS	6.78 ± 0.03^abc^	11.44 ± 0.85^bc^	7.31 ± 0.50^c^	44.00 ± 3.60^e^	65.07 ± 1.63^a^
50GM:50CS	6.56 ± 0.06^d^	12.70 ± 1.55^ab^	37.53 ± 2.68^a^	77.67 ± 4.04^b^	66.65 ± 5.53^a^
50GM:50AS	6.89 ± 0.09^a^	11.20 ± 2.10^bc^	30.32 ± 2.14^a^	21.67 ± 1.53^f^	49.33 ± 2.66^b^
25GM:75CS	6.29 ± 0.03^e^	12.73 ± 0.85^ab^	38.51 ± 1.81^a^	54.67 ± 4.04^d^	64.51 ± 1.70^a^
25GM:75AS	6.85 ± 0.02^ab^	8.45 ± 0.70^bc^	10.16 ± 1.14^bc^	134.67 ± 2.89^a^	72.70 ± 0.92^a^
0GM:100CS	6.68 ± 0.02^cde^	10.61 ± 1.46^bc^	35.53 ± 1.81^a^	63.67 ± 1.53^c^	43.82 ± 4.84^b^
0GM:100AS	6.85 ± 0.04^a^	11.80 ± 0.93^bc^	7.81 ± 0.92^bc^	69.33 ± 0.58^bc^	43.20 ± 1.49^b^

a Different letters within the same column indicate significant differences among the films according to Tukey’s test (*p* < 0.05).

All films exhibited surface pH values ranging from 6.2 to 6.9 (Table 6), with some formulations approaching the pH of the oral mucosa (≈6.8). This is a favorable characteristic because it may help reduce the risk of mucosal irritation during oral administration.^23,45^ Films formulated with GM and CS were slightly more acidic than those containing AS, with pH differences ranging from 0.21 to 0.56 units. The greatest difference was observed for the 25:75 formulations. Shin et al.^2^ reported that lower surface pH values may accelerate film disintegration and consequently promote faster release of incorporated active compounds.

Regarding the water contact angle (Table 6), films prepared with both starches exhibited hydrophilic characteristics, as all measured values were below 90°.^46^ In general, combining GM with the starches increased the contact angle and therefore reduced film wettability, except for the 50GM:50AS (49.33°) and 75GM:25CS (49.61°) formulations, which showed lower values. Higher contact angles may result from stronger intermolecular interactions among the polymer chains, particularly hydrogen bonding, which can reduce the availability of hydrophilic groups for interaction with water.^23,47,48^ It should also be noted that the water affinity of the polymer matrix may influence the release of subsequently incorporated active compounds, as more wettable ODFs generally allow faster water penetration and may consequently disintegrate and release the active ingredient more rapidly.^23^

A smaller contact angle indicates greater surface wettability, whereas a larger contact angle reflects lower wettability. Therefore, the positive association between contact angle and disintegration time indicates that less wettable films tended to disintegrate more slowly. Conversely, formulations with smaller contact angles generally promoted faster initial water spreading and penetration, contributing to shorter disintegration times. However, water absorption may induce swelling and partial dissolution immediately after droplet deposition. Thus, the measured values should be interpreted as indicators of initial surface wettability rather than equilibrium contact angles, because the dynamic response of these hydrophilic matrices may prevent the establishment of true equilibrium conditions.

Contact angle alone, however, did not fully explain the disintegration behavior. For example, 25GM:75CS and 25GM:75AS exhibited statistically similar contact angles, whereas their disintegration times differed significantly (54.67 and 134.67 s, respectively; *p* < 0.05). This result suggests that, despite comparable initial surface wettability, the GM/AS matrix formed a more resistant or densely organized network, which limited water penetration into the bulk material and delayed film perforation and disintegration.

Thus, formulations containing GM and starch that exhibited lower contact angles also showed shorter disintegration times, namely 41.00 s for 75GM:25CS and 21.67 s for 50GM:50AS. A similar relationship was reported by Garcia et al.^47^ and Garcia et al.^24^

Overall, most of the films disintegrated within approximately 60 s (Table 6). Currently, no standardized quality-control test has been established specifically for ODFs. The Brazilian Pharmacopoeia establishes a maximum disintegration time of 30 min for film-coated tablets and defines disintegrated units as those converted into a soft mass without a palpable core during testing.^49^ In contrast, the U.S. Food and Drug Administration (FDA) recommends a disintegration time of 30 s for orally disintegrating and lyophilized tablets, whereas the European Medicines Agency (EMA) allows up to 180 s^46^ Considering this range of 30–180 s, the disintegration times obtained for the developed films fall within the values commonly adopted for rapidly disintegrating oral dosage forms.

It is important to note that, during the in vitro disintegration test, only one side of the film was in contact with the aqueous medium. This condition does not reproduce the physiological environment of the oral cavity, where the film may be simultaneously moistened by saliva and exposed to tongue contact, pressure, and movement, all of which can accelerate disintegration.^46^ Therefore, this method provides a comparative measure of film disintegration under controlled conditions rather than a direct simulation of oral performance. Accordingly, the obtained values were interpreted as a screening tool for comparing the formulations. Disintegration times under in vivo conditions may be shorter.

As shown in Table 6, the disintegration time did not depend exclusively on the polymer composition of the film matrices, as a nonlinear behavior was observed. The shorter disintegration times of 100GM:0 and 50GM:50AS may be associated with their relatively low contact angles, which favored the initial spreading and penetration of water. In contrast, 25GM:75AS combined the highest contact angle with the longest disintegration time, indicating more limited initial wetting and a polymer network that was more resistant to water penetration. However, contact angle alone did not fully explain the behavior of all formulations, because disintegration also depended on film thickness, moisture content, water uptake, and the integrity and packing of the polymer network. Therefore, the observed results reflect the combined effects of surface wettability and the internal structural organization of the material.

The mechanical properties of the films are also presented in Table 6. Tensile strength reflects the resistance of a film to rupture under tension, whereas elongation at break represents its ability to stretch before failure.^2^ These properties are strongly influenced by polymer composition, plasticizer content, and the concentration of the film-forming materials.

The films exhibited tensile strength values ranging from 8.45 to 16.19 MPa. The highest value was observed for the control formulation (100GM:0), whereas the lowest was recorded for 25GM:75AS. Overall, starch incorporation did not produce a consistent trend in tensile strength, although higher AS proportions tended to reduce film strength.

Elongation at break varied considerably among the formulations, ranging from approximately 7.31% to 38.51%. Films containing CS generally exhibited higher elongation at break, particularly 25GM:75CS, which showed the highest value. In contrast, films with higher AS contents tended to be less extensible, indicating a more brittle structure.

These results suggest that CS contributed to the greater flexibility of the GM-based films, possibly because of differences in molecular organization and intermolecular interactions within the polymer matrix. Conversely, AS may have promoted a more rigid network, reducing chain mobility and limiting film extensibility.

Structural differences between the starches may help explain this behavior. Variations in amylose content can affect intermolecular interactions and packing density within the film matrix.^50^ A more compact structure may restrict molecular mobility and consequently reduce elongation at break. From a practical perspective, greater flexibility may improve handling and reduce the risk of fracture during processing and packaging, whereas more rigid films may be advantageous when greater dimensional and mechanical stability is required.

However, amylose content alone does not fully explain the mechanical behavior of the films. Flexibility may also be influenced by factors such as polymer compatibility, sorbitol distribution, residual moisture, chain wettability, and polymer-chain organization during drying. This is particularly evident for 50GM:50AS, which exhibited high elongation at break, suggesting that the 1:1 polymer ratio promoted a more favorable balance between intermolecular interactions and chain mobility. Nevertheless, this formulation also showed pronounced stickiness during demolding. Stickiness primarily reflects surface adhesion and may be influenced by retained water, sorbitol mobility, and polymer–surface interactions. In contrast, the lower elongation at break observed for several AS-containing films is consistent with restricted chain mobility within the bulk matrix. Thus, a relatively rigid internal network may coexist with a more adhesive surface.

Figure 5 presents the moisture content, moisture loss, moisture uptake, and thickness of the films. The 50GM:50AS formulation exhibited the highest moisture content among the evaluated samples (Figure 5C), which may have contributed to its greater elongation at break because retained water can act as a secondary plasticizer within the polymer matrix. The GM/AS formulations also exhibited greater moisture uptake, ranging from approximately 60 to 80%, whereas the GM/CS formulations showed lower values, ranging from approximately 10 to 25%. These differences indicate that polymer composition strongly influences water affinity and may consequently affect film handling, storage stability, and disintegration behavior.^45^

**Figure 5:**
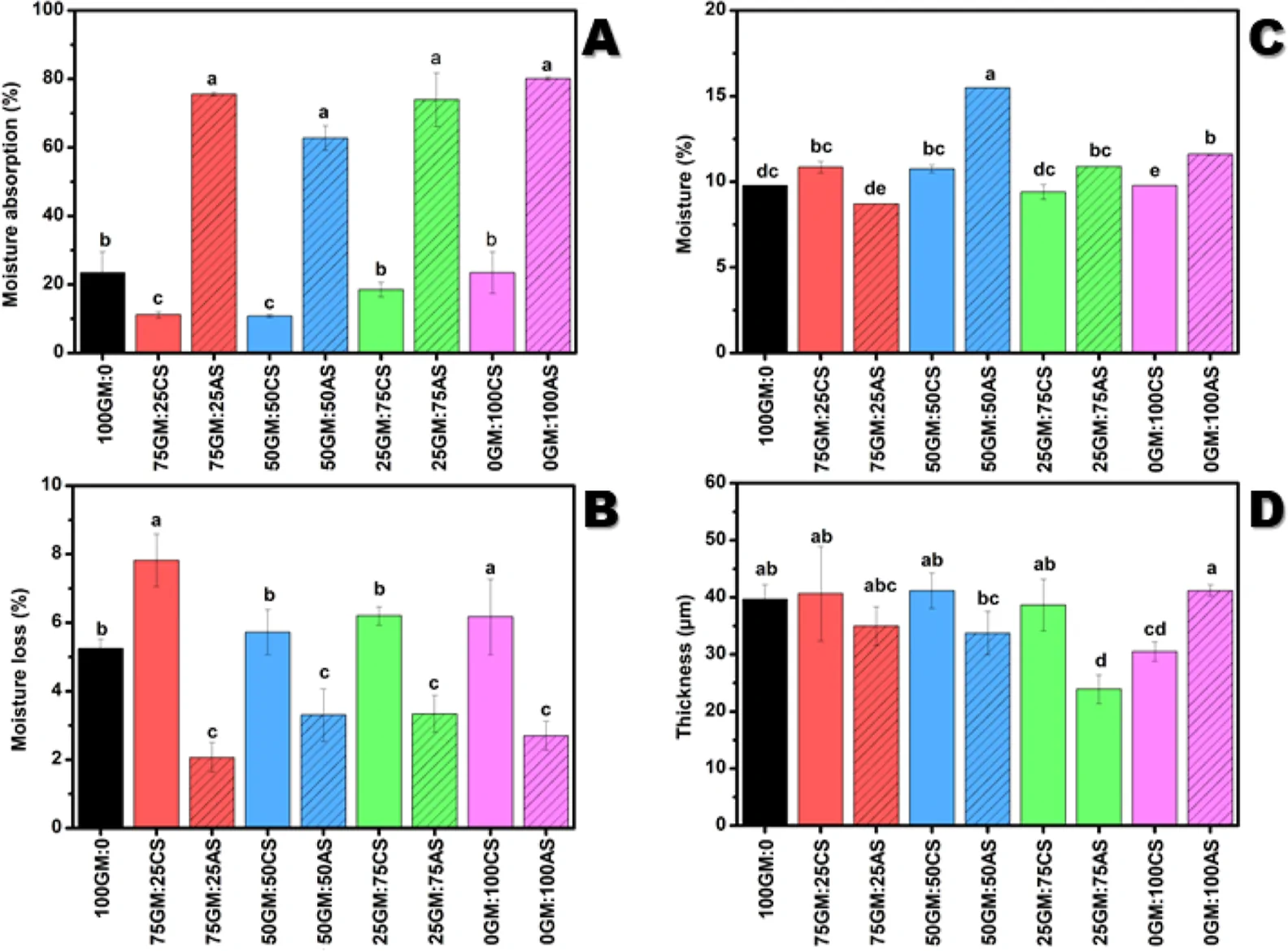
Moisture uptake (A), moisture loss (B), moisture content (C), and thickness (D) of films prepared with different GM/CS and GM/AS ratios. Different lowercase letters above the bars indicate significant differences among the films according to Tukey’s test (*p* < 0.05).

Regarding moisture loss (Figure 5B), films containing GM and CS exhibited a greater capacity for water loss. The 25GM:75CS and 50GM:50CS formulations showed the highest values, approximately 6%, similar to that of the GM control film. In contrast, the incorporation of AS reduced moisture loss to an average of 2.85%, with no significant differences among the different GM/AS ratios. According to Sowjanya and Rao, films with a greater tendency to lose moisture may become more rigid and brittle during storage. Therefore, the higher moisture loss observed for the GM/CS films may affect their long-term flexibility and handling stability.

These results have practical implications for film handling and oral performance. Formulations with greater moisture uptake may retain more water within the polymer matrix, which can contribute to increased surface tackiness during demolding. Conversely, films with lower moisture uptake generally exhibited better handling characteristics.^43^ Moisture-related properties may also influence oral disintegration because water uptake promotes matrix hydration and film disruption. However, this behavior is also governed by surface wettability and polymer-network organization.^51^

As shown in Figure 5D, the films exhibited relatively small variations in thickness, ranging from 23.91 to 41.17 μm. The 25GM:75AS and 0GM:100CS formulations were the thinnest, with thicknesses of 23.91 and 30.50 μm, respectively. However, thickness should be interpreted together with polymer composition, hydration behavior, wettability, and polymer-network organization rather than as an isolated determinant of film performance.

### 3.6 FT–IR

Figure 6 shows the FTIR spectra of films prepared with different GM/CS and GM/AS ratios. The spectra retain the characteristic absorption bands of the precursor materials (galactomannan, starch, and sorbitol).^9,23^

**Figure 6:**
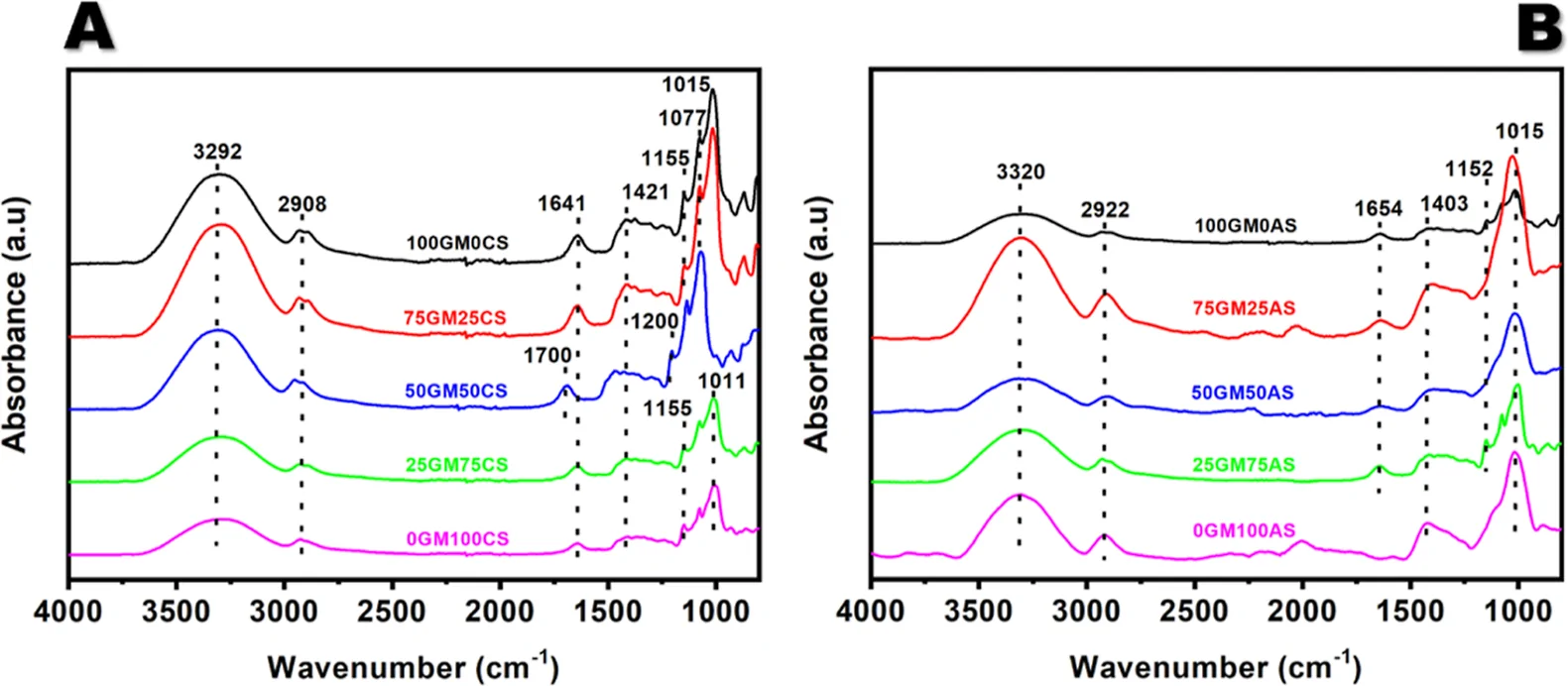
FT-IR spectra of films prepared with different GM/CS (A) and GM/AS (B) ratios.

The broad band observed in the 3000–3600 cm^–1^ region was attributed to O–H stretching vibrations.^9,23^ The absorption bands between 2800 and 3000 cm^–1^ were assigned to C–H stretching vibrations.^9^ The band near 1640 cm^–1^ was associated with H–O–H bending vibrations of water molecules adsorbed by the polymers, whereas the bands in the 1200–900 cm^–1^ region were related to coupled stretching vibrations of C–C–O, C–OH, and C–O–C bonds.^52^

Table S2 presents the main FTIR absorption bands, wavenumber shifts, relative band areas, and corresponding functional-group assignments for the pure and blended GM/CS and GM/AS films. Overall, the blended films exhibited wavenumber shifts relative to films prepared from a single polysaccharide, suggesting interactions between the polymer components. Because these shifts involved bands associated with hydroxyl groups, the observed interactions were likely mediated by hydrogen bonding.

The largest shifts were observed for the 1:1 GM/starch formulations. In 50GM:50CS, the O–H stretching band was observed at 3325 cm^–1^, whereas in films composed solely of GM and CS, this band occurred at approximately 3294 cm^–1^. In 50GM:50AS, the O–H stretching band was recorded at 3315 cm^–1^, while the corresponding bands in films prepared exclusively from GM and AS were observed at 3320 and 3311 cm^–1^, respectively. Additional evidence of interactions between the polysaccharides was observed in the region associated with the glycosidic backbone. In 50GM:50CS, the corresponding band appeared at 1072 cm^–1^, whereas in the GM and CS films, the bands were recorded at 1020 and 1011 cm^–1^, respectively. A similar shift was observed for 50GM:50AS, in which the corresponding band shifted from approximately 1020 to 1072 cm^–1^.

Relative integration of the FTIR spectra provided quantitative support for hydrogen-bonding interactions. Lower relative areas of the bands in the 3000–3600 cm^–1^ region may indicate stronger interactions between the precursor polymers.^53^ Among all formulations, 50GM:50AS exhibited the lowest relative band area (35.75%), followed by 0GM:100AS (41.28%) and 50GM:50CS (44.42%). In contrast, 75GM:25CS (53.35%) and 75GM:25AS (53.63%) showed the highest relative areas.

The higher relative band areas observed for the 75GM:25CS and 75GM:25AS formulations may indicate a greater availability of hydroxyl groups for additional hydrogen-bonding interactions, including interactions with water molecules. Conversely, the lower relative areas observed for the 50:50 formulations suggest stronger intermolecular interactions within the polymer matrix, consistent with increased hydrogen bonding between GM and starch. These findings agree with the wavenumber shifts observed in the O–H stretching region and support the occurrence of polymer–polymer interactions in the blended films.

### 3.7 Surface Morphology of Films

Figure 7 presents the SEM micrographs of films prepared with different GM/CS and GM/AS ratios, together with their respective controls. The film composed solely of GM exhibited surface irregularities, including light-colored aggregated regions. Starch incorporation altered this morphology, generally producing more uniform surfaces and reducing the visible heterogeneity of the GM matrix. These changes may reflect differences in polymer dispersion and network consolidation during film formation.^9,54^

**Figure 7:**
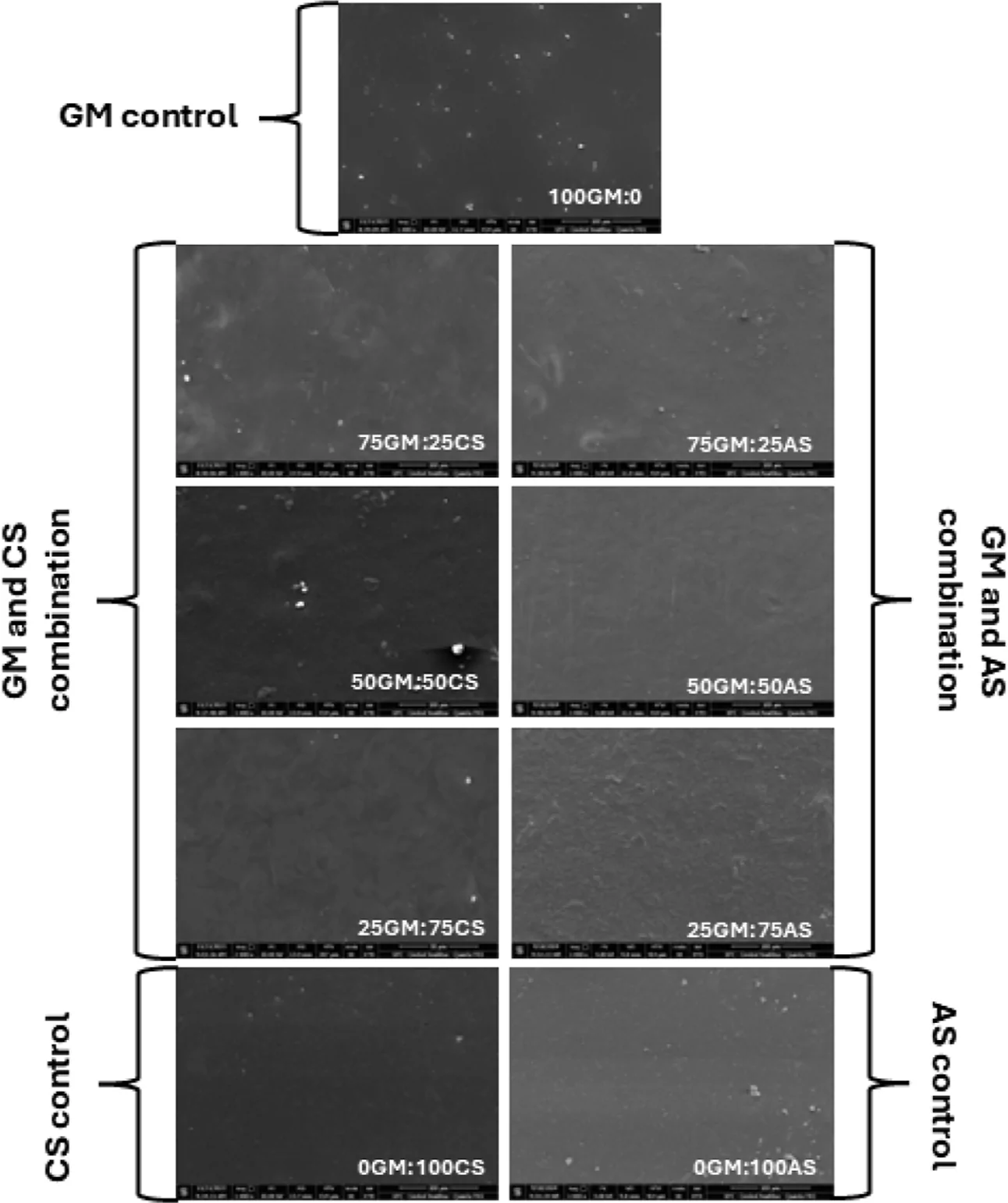
SEM micrographs of films prepared with different GM/CS and GM/AS ratios at 1000× magnification.

The observed morphological differences were consistent with variations in the mechanical properties, disintegration time, and contact angle of the films. The 75GM:25CS and 50GM:50AS formulations exhibited comparatively smoother surfaces, suggesting improved morphological compatibility between the polysaccharides at these ratios. These formulations also showed relatively short disintegration times, which is favorable for ODF applications.

Although the 25:75 formulations exhibited fewer visible aggregates, their surfaces appeared rougher and less uniform. This morphology may be associated with a more heterogeneous polymer-network organization and may have contributed to the distinct functional behavior observed for these films. However, SEM images alone do not confirm residual starch granules or phase-separated domains.

These composition-dependent morphological differences may help explain the distinct functional behavior of the films. Smoother surfaces may indicate improved network consolidation, which could favor more uniform stress distribution and surface wettability. In contrast, rougher structures may contribute to greater heterogeneity in mechanical performance and disintegration behavior. However, SEM images alone do not confirm the presence of residual starch granules or phase-separated domains.

### 3.8 Correlation between the Physical and Mechanical Parameters Analyzed

Figure 8 presents the principal component analysis (PCA), showing the relationships among the evaluated variables in Figure 8A, including contact angle, in vitro disintegration time, moisture loss, moisture uptake, surface pH, mechanical properties (tensile strength and elongation at break), and thickness, as well as the distribution of the film formulations in Figure 8B. The loading values of the variables for PC1 and PC2 are presented in Supplementary Table S3.

**Figure 8:**
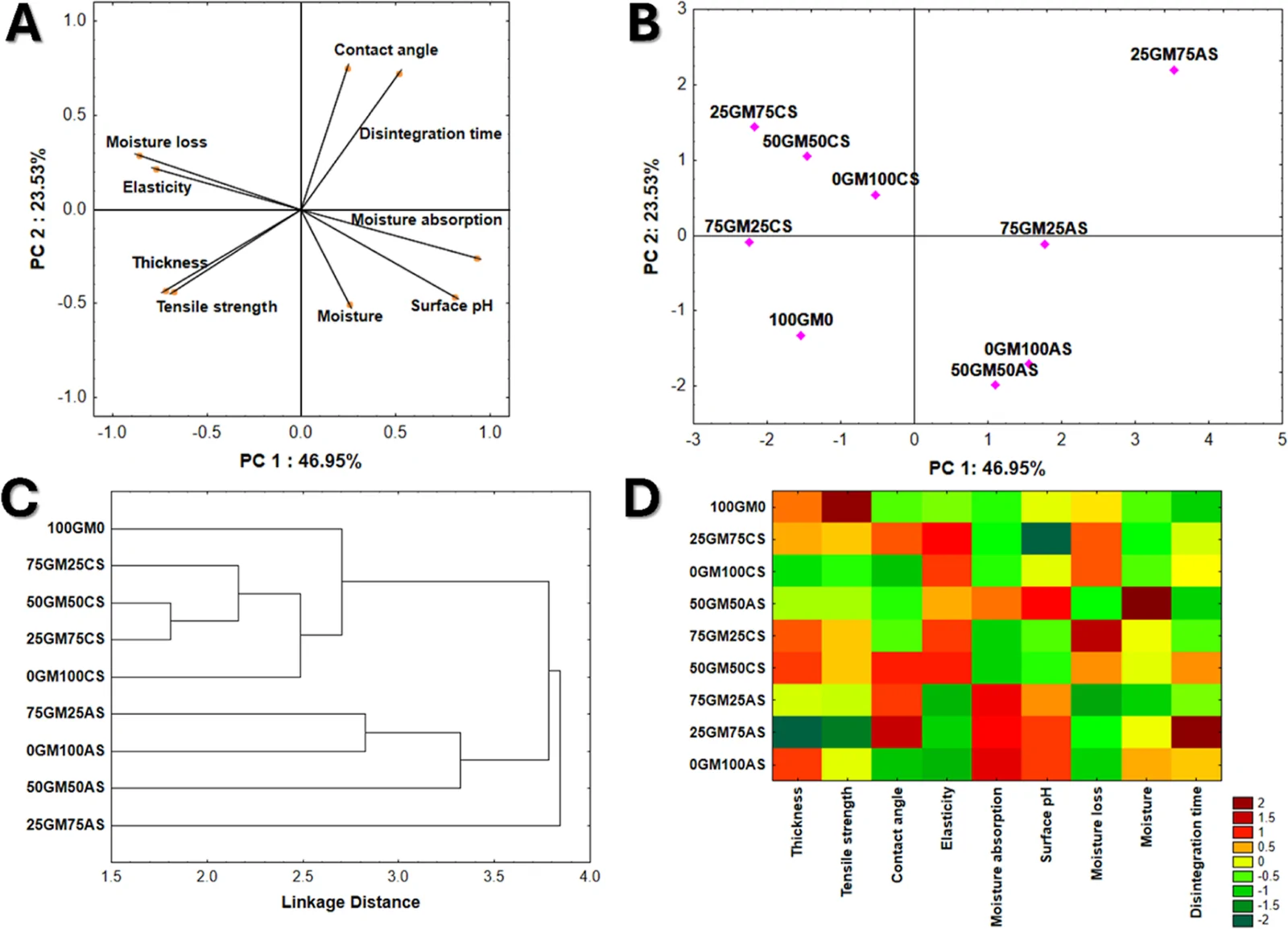
Principal component analysis (PCA) of the film formulations. (A) Loading plot (PC1 × PC2) of the evaluated physical and mechanical properties. (B) Score plot (PC1 × PC2) of the film formulations. (C) Hierarchical cluster analysis. (D) Two-way hierarchical cluster analysis (heatmap).

The principal components PC1 and PC2 together accounted for 70.48% of the total variance. In the PC1–PC2 loading plot shown in Figure 8A, contact angle and disintegration time were positively associated, indicating that less wettable films tended to require longer disintegration times. Elongation at break and moisture loss were also positively associated in quadrant 2, suggesting that variations in water content may influence film flexibility. In quadrants 3 and 4, thickness and tensile strength were positively associated with each other and negatively associated with most of the other evaluated variables. According to the loading values presented in Table S3, PC1 was mainly associated with moisture uptake, moisture loss, elongation at break, tensile strength, thickness, and surface pH, whereas PC2 was primarily influenced by disintegration time and contact angle.

Figure 8B shows that films containing AS and GM were clearly separated from those containing CS and GM, as the two groups were positioned on opposite sides of PC1. In addition, the 100GM:0 control film was located closer to the GM/CS formulations, indicating greater similarity in the overall pattern of evaluated properties.

The hierarchical cluster analysis (Figure 8C) was consistent with the PCA results, separating the formulations into groups according to polymer composition. Formulations containing higher CS proportions tended to cluster together, whereas AS-rich films formed a distinct group, highlighting the influence of starch type on the overall pattern of film properties.

The two-way hierarchical cluster analysis of the evaluated film properties is shown in Figure 8D. The control formulations and 75GM:25CS were associated with lower contact angles and shorter in vitro disintegration times, indicating greater initial surface wettability. In contrast, formulations containing higher AS proportions tended to exhibit greater moisture uptake and lower moisture loss, which may reflect differences in polymer-network organization. Together, these properties help explain the distinct performance of the formulations as potential ODF matrices.

### 3.9 Mechanistic Interpretation of Structure–Function Relationships

Figure 9 summarizes the proposed structure–function relationships linking polymer composition to the macroscopic performance of the films. The high molar mass and relatively low degree of galactose substitution of GM (M/G = 2.53:1) may favor chain entanglement and pseudoplastic behavior in solution, making GM a major contributor to network formation. As starch content increased, the predominance of the entangled GM network decreased, shifting the rheological behavior toward dilatancy and lowering the consistency index. Although this reduction occurred for both starches, AS-containing systems generally retained higher consistency than the corresponding CS-containing systems, likely because of the higher amylose content of AS. These rheological differences were also reflected in film morphology: intermediate compositions, particularly 75GM:25CS and 50GM:50AS, exhibited smoother and more homogeneous surfaces, whereas starch-rich films showed less uniform and apparently less cohesive structures.

**Figure 9:**
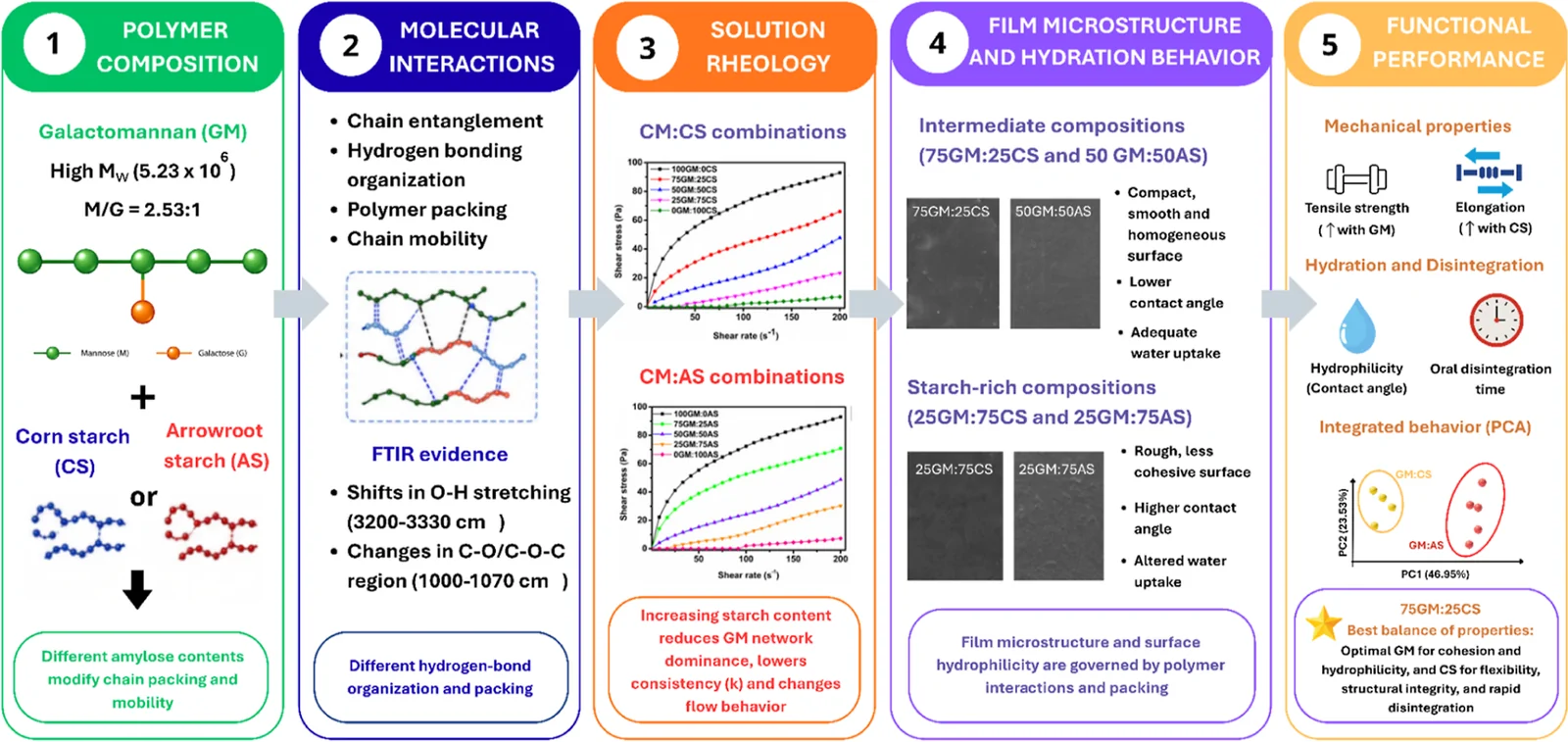
Proposed structure–function relationships in GM/CS/AS orally disintegrating films. Polymer composition controls chain entanglement, hydrogen-bond organization, molecular packing, and solution rheology. These features determine film microstructure and water interaction, which ultimately govern tensile strength, elongation, and oral disintegration.

At the molecular level, FTIR analysis indicated composition-dependent changes in hydrogen-bond organization and polysaccharide-chain packing. These differences may have influenced polymer–water interactions and were reflected in the contact angle and disintegration behavior of the films. Amylose content also affected the mechanical response. The higher amylose content of AS may have favored closer chain packing and restricted molecular mobility, thereby contributing to lower elongation at break. In contrast, CS-containing films maintained greater chain mobility and generally exhibited higher elongation at break.

The PCA integrated these trends by revealing the positive association between contact angle and disintegration time and the separation between the GM/AS and GM/CS formulations, reflecting differences in the overall pattern of properties associated with starch type and amylose content.

Overall, the sequence linking polymer composition, solution rheology, film microstructure, hydration behavior, and mechanical and disintegration performance helps explain why 75GM:25CS exhibited the most balanced technological profile among the evaluated formulations. Although 100GM:0 and 50GM:50AS showed shorter disintegration times, 75GM:25CS combined relatively rapid disintegration with adequate tensile strength, high elongation at break, favorable wettability, and better handling and demolding characteristics.

This study should be considered a preliminary investigation aimed at screening polymeric matrices with properties suitable for ODF applications, as no active compound was incorporated at this stage. The identification of 75GM:25CS as the formulation with the most balanced technological properties provides a rational basis for continuing this research through the incorporation of an active compound and subsequent evaluation of its release and functional performance.

## 4 Conclusion

This study demonstrated that the functional performance of galactomannan–starch films was governed by the combined effects of polymer composition, solution rheology, network organization, and polymer–water interactions. The high molar mass of GM increased solution consistency and contributed to the formation of cohesive film matrices. CS generally improved film flexibility, whereas AS tended to promote a more compact and rigid structure, particularly at higher proportions, which may also have contributed to slower disintegration. However, blend behavior was composition-dependent, as demonstrated by the comparatively high elongation at break of 50GM:50AS.

Film disintegration was not governed by wettability alone but by the balance among initial water spreading, water penetration, and polymer-network integrity. Among the evaluated formulations, 75GM:25CS exhibited the most balanced combination of mechanical strength, elongation at break, wettability, handling characteristics, and relatively rapid disintegration, supporting its selection as the most promising matrix for further development.

Because this study was conducted as a preliminary screening of polymeric matrices without the incorporation of an active compound, future work should evaluate the incorporation of pharmaceutical or nutraceutical bioactive compounds, release behavior, and storage stability. Further studies should also assess mucoadhesion, bioaccessibility, and film performance under more physiologically representative oral conditions.

## Supplementary Material



## Notes

DSC thermal transition temperatures, FTIR spectral data and relative band areas, PCA loading values, and ^1^H NMR spectra of corn and arrowroot starches (PDF)
