# Supplementary material for: Structure–Function Relationships in Galactomannan Starch Films for Fast-Dissolving Applications: Effects on Physicochemical and Mechanical Properties
Source: ACS Omega. 2026 Aug 28;11(36):54153–68. doi: 10.1021/acsomega.6c04905 (PMC13584513; doi:10.1021/acsomega.6c04905)
Supplement: Supplementary file 1 [file ao6c04905_si_001.pdf]

Structure-function relationships in galactomannan starch films for fast-dissolving applications: effects on physicochemical and mechanical properties

Lorena M. F. Sampaio[1], Matheus X. Oliveira[2], Venícios G. Sombra[2], Judith P. A. Feitosa[2], Nayanne L. dos S. Ferreira[1], Maria do S. R. Bastos[3], Sara J. Marciano[2], Diego Lomonaco[2], Amélia R. N. Lima[1], Lara Mota Soares[1], Crisiana de A. Nobre[1], Kaliana S. Eça[1], Thomas Karbowiak[4], Luciana de S. Oliveira[1*]

[1]Departamento de Engenharia de Alimentos, Universidade Federal do Ceará, Avenida Mister Hull, s/n, Campus Pici, 60455-760, Fortaleza, Ceará, Brasil.

[2]Departamento de Química Orgânica e Inorgânica, Universidade Federal do Ceará, Avenida Mister Hull, s/n, Campus Pici, 60455-760, Fortaleza, Ceará, Brasil.

[3]Embrapa Agroindústria Tropical, Rua Doutora Sara Mesquita, 2270, Planalto do Pici, 60511-110, Fortaleza, Ceará, Brasil.

[4]Université Bourgogne Europe, Institut Agro, INRAE, UMR PAM (Procédés Alimentaires et Microbiologiques), Bâtiment EPICURE, 1 Esplanade Erasme, 21000, Dijon, France.

*Corresponding author
Luciana de Siqueira Oliveira
luciana.oliveira@ufc.br

Supplementary material

Table S1. Onset (Tonset), peak (Tpeak), and endset (Tendset) temperatures of the endothermic transitions obtained from the DSC curves of the polysaccharides.

Sample	Tonset (ºC)	Tpeak (ºC)	Tendset (ºC)
CS	63.60	68.43	71.85
AS	77.70	81.11	85.03
GM	-	-	-

GM: no endothermic transition observed between 25 and 100 °C.

Table S2. Main FTIR bands, wavenumber, relative band areas, and functional-group assignments for pure and blended GM:CS and GM:AS films.

Bands (cm^{-1})	Ratio	GA:CS		Bands (cm^{-1})	Ratio	GA:AS		Functional group
		A	%A			A	%A	
3292	100:0	0.38	54.14	3320	100:0	0.38	54.14	-OH
3310	75:25	1.43	53.35	3315	75:25	0.38	53.63	
3325	50:50	0.98	44.42	3315	50:50	0.40	35.75	
3325	25:75	0.54	50.42	3307	25:75	0.67	49.33	
3294	0:100	0.45	50.90	3311	0:100	0.78	41.28	
2922	100:0	0.02	3.00	2922	100:0	0,02	3.00	-C-H
2930	75:25	0.07	2.47	2908	75:25	0.01	2.09	
2911	50:50	0.05	2.46	2951	50:50	0.04	4.00	
2951	25:75	0.03	2.57	2928	25:75	0.04	2.80	
2925	0:100	0.03	3.19	2918	0:100	0.09	4.57	
1641	100:0	0.02	3.45	1654	100:0	0.02	3.45	-OH
1648	75:25	0.07	2.70	1642	75:25	0.02	2.80	
1690	50:50	0.05	2.18	1636	50:50	0.03	2.30	
1690	25:75	0.03	3.28	1646	25:75	0.03	2.05	
1648	0:100	0.02	2.52	NV*	0:100	NV	NV	
1020	100:0	0,18	24.87	1020	100:0	0,18	24.87	-C-O-H; C-C-O; -C-O-C;
1031	75:25	0.67	25.25	1016	75:25	0.17	25.37	
1072	50:50	0.53	24.05	1072	50:50	0.34	29.71	
1002	25:75	0.30	26.12	1002	25:75	0.33	24.61	
1011	0:100	0.23	26.40	1020	0:100	0.44	23.15	

NV: no vibration in this region

Table S3. Loading values of the variables for the first two principal components (PC1 and PC2) obtained from principal component analysis (PCA).

Variables	PC1	PC2
Thickness	-0.676	-0.434
Tensile strength	-0.720	-0.429
Elasticity	-0.774	0.217
Moisture absorption	0.930	-0.257
Moisture loss	-0.860	0.290
Moisture	0.251	-0.503

Disintegration time	0.517	0.724
Surface pH	0.812	-0.462
Contact angle	0.242	0.748

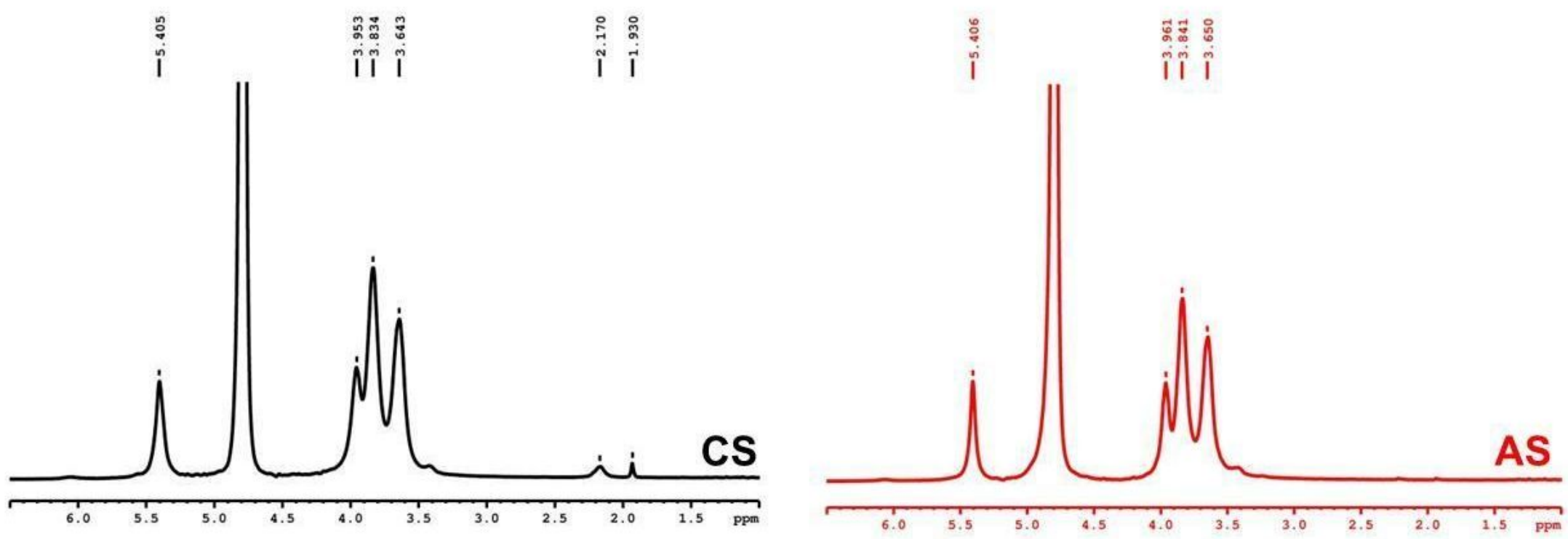

Figure S1. ^{1}H-NMR spectrums (D_2O, 70 ºC, 500 MHz) of CS and AS.
